# Detection of Chromosome X;18 Breakpoints and Translocation of the Xq22.3;18q23 Regions Resulting in Variable Fertility Phenotypes

**DOI:** 10.1155/2012/681747

**Published:** 2011-11-21

**Authors:** Attila Szvetko, Nicole Martin, Chris Joy, Andrea Hayward, Bob Watson, Andrew Cary, Stephen Withers

**Affiliations:** ^1^Genetics Department, Genesis Clinical Genetics, Suite 3 Allamanda Medical Centre, 25 Spendelove Street, Southport, Qld 4215, Australia; ^2^Cytogenetics Laboratory, Queensland Fertility Group, 1st Floor, 225 Wickham Terrace, Brisbane, Qld 4000, Australia; ^3^Genetics Department, QML Pathology, 11 Riverview Place, Metroplex on Gateway, Murarrie, Qld 4172, Australia; ^4^Brisbane IVF Clinic, Queensland Fertility Group, 125 Flockton Street, Everton Park, Brisbane, Qld 4053, Australia; ^5^Gold Coast IVF Unit, Fertility Gold Coast, Pindara Private Hospital, 13 Carrara Street, Benowa, QLD 4217, Australia

## Abstract

We describe a familial pattern of gonosomal-autosomal translocation between the X and 18 chromosomes, balanced and unbalanced forms, in male and female siblings. The proposita was consulted for hypergonadotropic hypogonadism. Karyotype analysis revealed a balanced 46, X, t(X;18)(q22.3;q23) genotype. The sister of the proband presented with oligomenorrhea with irregular menses and possesses an unbalanced form of the translocation 46, X, der(X), t(X;18)(q22.3;q23). The brother of the proband was investigated and was found to possess the balanced form of the same translocation, resulting in disrupted spermatogenesis. Maternal investigation revealed the progenitor karyotype 46, X, t(X;18)(q22.3;q23). Maternal inheritance and various genomic events contributed to the resultant genotypes. Primary infertility was initially diagnosed in all progeny; however, the male individual recently fathered twins. We briefly review the mechanisms associated with X;18 translocations and describe a pattern of inheritance, where breakpoints and translocation of the Xq22.3;18q23 regions have resulted in variable fertility.

## 1. Introduction

Numerous X-chromosomal translocations have been reported in the literature. The phenotypic manifestations of these translocations depend on several factors including the nature of the translocation: X-autosomal, Y-autosomal, or X-Y chromosomal exchange, as well as the pattern of inheritance, segregation, and gene inactivation [1, 2]. Most fertility mutations affect gonad function, but it is also apparent that a number of fertility genes are yet to be realised [3]. Historically, autoradiographical analyses delineated common patterns of X-chromosomal replication and inactivation of reciprocal autosomes [4, 5]. X-autosome translocations can effect fertility where chromosomal changes result in inactivation of genes governing reproduction [6]. Gonosomal-autosomal translocations are distinct aberrations, which involve an autosome and/or gonosome (sex chromosome). These variations involve site-specific loss or gain of genetic material with the latter type giving rise to trisomies, the former being monosomies. Translocations of this class can be complex [7]. Studies examining X-chromosome deletions have predicted that Xq aberrations within the Xq13–Xq27 region can result in premature gonad failure [8]. X-autosome translocation causing gonadal dysgenesis with bilateral streak gonads as well as aberrant ovarian and sex development has been demonstrated by numerous studies [9, 10]. Translocations involving the long arms of the X-chromosome and several autosomes (X; 1–4, 6–9, 11, 12, 14, 15, 17, 19, 21, and 22), resulting in various degrees of gonad dysfunction, have also been reported [8]. Recently, it has become evident that breakpoints in the critical region on Xq21 play a significant role in premature ovarian failure (POF) due to effects on flanking genes [11]. Reciprocal translocations between autosomes and gonosomes are irregularities that appear to contribute significantly to primary infertility. In the current paper, we describe a case of X;18 translocation in an Australian kindred, resulting in various degrees of infertility in both male and female siblings. 

## 2. Case Presentation

**Figure 1:**
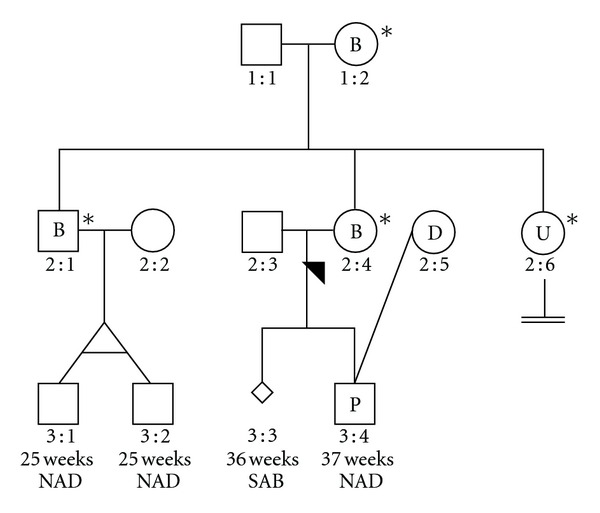
Pedigree. *Affected individual detected. B: balanced translocation detected; 46, X, t(X;18)(q22.3;q23). U: Unbalanced translocation detected; 46, X, der(X), t(X;18)(q22.3;q23). D: donor—individual 2 : 4 carried pregnancy 3 : 4 using donor egg (from individual 2 : 5) and partner's sperm (from individual 2 : 3). P: Pregnancy resulted from donor egg (from individual 2 : 5). SAB: spontaneous abortion (individual 3 : 3). NAD: no abnormalities detected.

The proposita is the second of three siblings (Figure 1; individual 2 : 4), conceived when the mother was 21 years of age. Individual 2 : 4 was first consulted at 33 years of age upon referral for suspected infertility. Initial investigations revealed hypergonadotropic hypogonadism (elevated gonadotrophins with low estradiol and progesterone), indicating poor ovarian function with ovarian resistance. Results were confirmed on several occasions, and premature menopause was diagnosed. Following a short course of ovulatory induction, this patient experienced an early pregnancy at age 36; however, that pregnancy resulted in spontaneous abortion at 36-week gestation (individual 3 : 3). Sporadic chromosomal abnormalities as well as both maternal and paternal meiotic errors may contribute to unsuccessful pregnancies of this type [12]. Recently, individual 2 : 4 successfully conceived a male child (Individual 3 : 4) following assisted fertility therapy using donor ovum (from Individual 2 : 5) and partner sperm (from individual 2 : 3). That pregnancy lasted 37 weeks, and the progeny (individual 3 : 4) was born without complication. The patient had been previously investigated for suitability to *in vitro *fertilization, which had on that occasion confirmed ovarian resistance. Both the proband and her partner underwent chromosome analysis. Individual 2 : 3 was determined karyotypically normal (46XY). For individual 2 : 4, cytogenetic studies revealed a 46XX genotype with balanced translocation of the long arms of an X-chromosome and the distal region of chromosome 18 (46, X, t(X;18)(q22.3;q23). The couple was informed of potentially viable pregnancy outcomes, and it was explained that the translocation was the most likely cause of the apparent ovarian failure, owing to a relative X-monosomy with partial inactivation of the second X-chromosome. The likelihood of the production of a chromosome 18 trisomy, leading to Edwards syndrome was discussed. Consultation regarding assisted reproductive technologies and preimplantation genetic diagnosis were undertaken. Analysis of chromosomal segregation patterns showed that of the 16 possible segregations (meiotic), only 2 could yield normal or balanced gametes. The risk of producing potential carrier siblings was discussed (Table 1).

The proband's brother, the first of three siblings (individual 2 : 1), was conceived at 19 years maternal age. He was aged 36 years at the time of initial presentation and was referred for fertility testing. On examination, the patient was phenotypically normal. Genomic analysis revealed a balanced X;18 translocation (46, Y, t(X;18)(q22.3;q23)). Concerns regarding the likelihood of infertility were discussed. X-autosome translocations are known to interfere with meiosis leading to disrupted spermatogenesis. It is known that most males and approximately half of all females with X-autosome translocations experience sterility [3]. Balanced reciprocal translocations are amongst the most common chromosomal abnormalities. Rearrangements usually result in sterility and/or a higher risk of chromosomal imbalance among offspring [13]. Seminal fluid analysis was conducted and individual 2 : 1 was found to have decreased motile sperm count (<25%), increased sluggish/nonprogressive sperm (90%), as well as elevated abnormal spermatozoa (99%). Overall, analysis showed quantitatively normal seminal fluid with reduced motility, with the increased presence of abnormal forms. The limited prospects of successful conception were discussed with the couple (individuals 2 : 1 and 2 : 2).

The sister of the proband is the youngest of the three siblings (individual 2 : 6) and was conceived at 25 years maternal age. She was referred with oligomenorrhea and irregular menstrual cycles (ranging from nil for 6 months to 5-week-long cycles). Cytogenetic analysis revealed a 46, X, der(X), t(X;18)(q22.3;q23) unbalanced translocation, that is, trisomic 18q karyotype. Other than the genotype, the subject was and continues to be phenotypically normal. She was further counselled regarding the decreased likelihood of successful pregnancy, and the translocation implications were explained.

The mother of the proband was consulted (individual 1 : 2). She had previously experienced three uncomplicated pregnancies at the ages of 19, 21, and 25 years. Chromosomal analysis revealed 46, X, t(X;18)(q22.3;q23) with a balanced reciprocal translocation between the long arms of one X-chromosome and chromosome 18 (18q). Cytogenetic analysis showed breakpoints at Xq22.3 and 18q23. This patient is phenotypically normal. Although Xq22 breakpoints may be associated with ovarian dysfunction, the patient reported no fertility issues and had previously produced three offspring, which resulted from uncomplicated full-term pregnancies. Pregnancies in such situations run the risk of unknowingly producing unbalanced gametes owing to their clinically silent presentation, which indeed was the case in this instance.

## 3. Discussion

In the current pedigree, we investigated three siblings conceived at maternal ages 19, 21, and 25 years, for chromosomal translocations, which were inherited from their mother who possesses a 46, X, t(X;18)(q22.3;q23) genotype. She carries a balanced form of the X;18 translocation, whilst two of the siblings carry the balanced form and one the unbalanced form. Pedigree analysis showed that the mother imparted the affected X-chromosome with the son carrying the altered complement. The proposita initially presented with a history of miscarriage and signs of ovarian deficiency. Genomic investigations determined the proband genotype to be a balanced 46, X, t(X;18)(q22.3;q23). The younger female sibling of the proband (Individual 2 : 6) was consulted, and although phenotypically normal, was found to possess an unbalanced translocation: 46, X, der(X), t(X;18)(q22.3;q23). The primary concern was reduced likelihood of successful conception. The eldest male sibling (individual 2 : 1) was tested and found to possess a balanced form of the X; 18 translocation (46, Y, t(X;18)(q22.3;q23)). This individual went on to recently father monozygotic twins (individuals 3 : 1 and 3 : 2). The twins were born at 25 weeks but show no obvious effects from the prematurity, or otherwise.

Autosomal translocations are relatively common; gonosome-autosome translocations with rearrangement of genes are rather unique. In the current case, a mix of both balanced and unbalanced translocation between the X- and 18 chromosomes has led to various degrees of fertility. Several interesting points punctuate the case; the mother's normal phenotype, the balanced/unbalanced inheritance pattern in the daughters, and the absence of clinically recognizable dysfunction other than described for the male sibling. The typical clinical characteristics of the unbalanced translocation (dysmorphic features, organ malformation, and retardation) are not present, and successful progeny for the male individual (individual 2 : 1) serves to distinguish the case. It is known that most males with this type of anomaly are completely spermtogenically impaired; however, this is not the case with the investigated male. Previous research has postulated that regions on the Y-chromosome as well as various autosomes may contribute to successful spermatogenesis in the presence of X-chromsomal aberrations [14]. Y-chromosomal compensation may be an explanation for the successful conception of the twins in this case.

In females, the phenotype depends on the position of the breakpoint as well as the functional status of the remaining X-chromosome. Approximately 75% of patients will have one active/one inactive X, while approximately 25% will have inactivation of the X-chromosome in only some cells. Females with an active X in all cells with the breakpoint not within any functional gene show a higher incidence (approximately 50%) of ovarian failure (mainly breakpoints within the Xq13–q26 critical region) [3]. In this case, the female siblings carry the balanced (individual 2 : 4) and unbalanced (individual 2 : 6) X;18 translocation with breakpoints at q22.3–q23. These changes have caused ovarian dysfunction. X-chromosomal activation/inactivation combined with variable gene expression can occur because translocated genetic material from the X-chromosome may or may not be reactivated once translocated [15]. Ovarian failure can result from X-chromosomal abnormalities, autosomal recessive genes causing various types of XX gonadal dysgenesis, and autosomal dominant gene expression. The exact X-chromosomal loci responsible can be difficult to localize, but there is evidence to suggest that in aggregate, these genes regulate ovarian maintenance. X-monosomies are known to result in accelerated germ-cell atresia [16]. The two known fertility genes mapped to the region Xq22.3–23 are the testis expressed 13A and 13B genes (TEX13A and TEX13B, resp.), and little is known about their functions. It is believed that TEX13A and TEX13B are involved in testicular development (spermatogonially expressed, germ-cell-specific genes) and it has been noted that they are well conserved across chimpanzee, dog, cow, mouse as well as *Homo sapiens* [1]. Although only limited information exists regarding this region, the X-chromosome has a predominant role in premeiotic stages of mammalian spermatogenesis, and an abundance of X-linked genes is known to be expressed in spermatogonia [17]. The only known ovarian-resistance/maintenance related gene within the area of interest Xq13–26 is the *Drosophila melanogaster* homologue *DIAPH2* (*DIA*). Aberrations in *DIAPH2 *are known to result in sterility in both male and female *Drosophila*, but its function in humans is not well defined [18]. *DIAPH2* regulates cytokinesis, which has a direct influence on oogenesis and follicle maturation. Two of the most prominent ovarian dysfunction genes with human homologues at the X and 18 chromosomes are the B-cell/lymphoma 2; *Bcl-2* (human homologue 18q21.3) and zinc finger protein, X-linked; *Zfx* (human homologue Xp21.3) genes. *Bcl-2* has been shown in knockout models to be involved in the accelerated atresia of primordial follicles, whilst deletion of *Zfx* results in reduced number of oocytes and general infertility by way of reduced germ cells [19, 20]. The ZFX gene in humans also shares characteristics with the zinc finger protein 711 gene (ZNF711), located Xq21.1-q21.2, which codes for a protein which acts as a transcription activator, presumably for fertility-related-gene regulatory elements [21]. In the current case, breakpoints and translocation of the Xq22.3;18q23 region are responsible for the characteristic pattern of presentation. Investigation and ongoing analysis of such anomalies may explain the often idiopathic nature of primary infertility syndromes.

## Figures and Tables

**Table 1:** Theoretical meiotic segregation patterns for translocation: 46, X, t(X;18)(q22.3;q23).

Segregation type	Cell 1	Cell 2
Alternate (result is balanced or normal)^a^	X + 18	der(X) + der(18)
Adjacent 1^b^	X + der(18)	der(X) + 18
Adjacent 2^b^	X + der(X)	18 + der(18)
Tertiary trisomy^b^	X + 18 + der(X)	der(18)
Tertiary trisomy^b^	X + 18 + der(18)	der(X)
Interchange trisomy^b^	der(X) + der(18) + X	18
Interchange trisomy^b^	der(X) + der(18) + 18	X
4 : 0^b^	X + der(X) + 18 + der(18)	—

^a^Normal or balanced meiotic segregation pattern.

^b^Abnormal theoretical meiotic segregation pattern.
